# Characterization and response of two potato receptor-like kinases to cyst nematode infection

**DOI:** 10.1080/15592324.2022.2148372

**Published:** 2022-11-23

**Authors:** Shiyan Chen, Melissa G. Mitchum, Xiaohong Wang

**Affiliations:** a School of Integrative Plant Science, Cornell University, Ithaca, NY, USA; b Department of Plant Pathology and Institute of Plant Breeding, Genetics & Genomics, University of Georgia, Athens, GA 30602, USA; c Robert W. Holley Center for Agriculture and Health, US Department of Agriculture, Agricultural Research Service, Ithaca, NY, USA

**Keywords:** CLE effector, CLE receptor, CLV1, *Globodera pallida*, *Globodera rostochiensis*, plant-parasitic cyst nematode, potato, potato cyst nematode (PCN), receptor-like kinase, RPK2

## Abstract

Plant-parasitic cyst nematodes (*Heterodera* and *Globodera* spp.) secrete CLAVATA3/EMBRYO SURROUNDING REGION-RELATED (CLE) effector proteins, which act as ligand mimics of plant CLE peptides to promote successful nematode infection. Previous studies of the Arabidopsis-beet cyst nematode (BCN; *H. schachtii*) pathosystem showed that Arabidopsis CLE receptors including CLAVATA1 (CLV1), CLV2, and RECEPTOR-LIKE PROTEIN KINASE 2 (RPK2) are required for BCN CLE signaling. Studies further revealed that nematode CLE signaling through GmCLV2 and StCLV2, an Arabidopsis CLV2 orthologue from soybean (*Glycines max*) and potato (*Solanum tuberosum*), respectively, is required for the soybean cyst nematode (SCN; *H. glycines*) and the potato cyst nematode (PCN; *G. rostochiensis*) to induce disease in their respective host plant. In this study, we identified and characterized two additional potato receptors, StRPK2 and StCLV1, homologues of Arabidopsis RPK2 and CLV1, for a role in PCN parasitism. Using promoter-reporter lines we showed that both *StRPK2* and *StCLV1* are expressed in the potato root but vary in their spatial expression patterns. Interestingly, *StRPK2* but not *StCLV1* was found to be expressed and upregulated at PCN infection sites. Nematode infection assays on *StRPK2*-knockdown lines revealed a decrease in nematode infection. Collectively, our results suggest that parallel CLE signaling pathways involving StCLV2 and StRPK2 are important for PCN parasitism and that manipulation of nematode CLE signaling may represent a viable means to engineer nematode resistance in crop plants including potato.

## Introduction

Potato (*Solanum tuberosum*) is the most important non-cereal food crop in the world. Effective pest and disease management is crucial for the sustainability of this crop. Potato cyst nematodes (PCN), *Globodera rostochiensis* and *G. pallida*, are devastating pathogens of potato that cause an estimated yield loss of 9% worldwide.^1^ PCN are obligate, endoparasitic nematodes that induce the formation of a feeding structure called a syncytium, a multinucleate and metabolically highly active structure of fused cells around the vasculature in potato roots.^2,3^ The syncytium serves as the sole nutrient source that sustains the growth and development of the nematode. It is now known that effector proteins delivered into the host root through the nematode’s stylet are the major signaling molecules responsible for the formation and maintenance of the syncytium.^3,4^

Effector genes encoding the CLAVATA3/EMBRYO SURROUNDING REGION-RELATED (CLE) proteins are among the best studied groups of effectors from plant-parasitic nematodes. Nematode *CLE* genes are widely distributed in various cyst nematode species, including PCN, the soybean cyst nematode (SCN; *Heterodera glycines*), and the beet cyst nematode (BCN; *H. schachtii*) that infects sugarbeet and other important vegetable crops.^3,5–11^ Previous studies have demonstrated that nematode-secreted CLE peptides function as mimics of plant CLE peptides to promote nematode parasitism.^6,8–12^ Plant CLEs are a large family of extracellular signaling peptides that stimulate receptor-mediated signaling pathways to modulate various developmental and physiological programs.^13,14^ Plant CLEs are grouped into either A-type or B-type peptides.^13,14^ The A-type peptides promote cell differentiation, the B-type peptides, on the other hand, inhibit differentiation of tracheary elements and promote procambial cell division. Leucine-rich repeat (LRR) receptor-like kinase (RLK) family members are the major players in perceiving CLE peptides. Of the 27 unique CLE peptides encoded by the Arabidopsis genome, CLAVATA3 (CLV3), an A-type peptide, is the best characterized member. Multiple receptor complexes, including CLV1, the LRR receptor-like protein CLV2 and the coreceptor CORYNE (CRN), BARELY ANY MERISTEM (BAM) receptor kinases, and BAM1 associated RECEPTOR-LIKE PROTEIN KINASE 2 (RPK2), are involved in perceiving the CLV3 peptide to regulate stem cell homeostasis in the shoot apical meristem.^14,15^ CLE signaling pathways also regulate many aspects of Arabidopsis root development. For example, many CLE peptides including CLE17 and CLE19 can induce premature termination of the root apical meristem (RAM) and a short root phenotype.^16^ It was recently showed that RPK2 mediates the root growth-arrest phenotype triggered by exogenous application of CLE17 or CLE19 peptide.^17^ Studies also revealed that CLV1 is involved in CLE40 signaling to regulate stem cell homeostasis in the distal region of the RAM as well as CLE3 signaling in roots under nitrogen-deficient conditions.^18,19^

The Arabidopsis-BCN pathosystem was initially used to investigate the mechanistic details of nematode CLE signaling during plant parasitism. Studies revealed that Arabidopsis receptors CLV2/CRN, CLV1, and RPK2 are involved in perceiving BCN CLEs and that this perception is required for successful nematode infection and syncytium development.^20,21^ Orthologues of these Arabidopsis receptors were identified from soybean (*Glycine max*), a host for SCN.^11^ All these CLE receptors were found to be expressed in SCN-induced syncytia in soybean roots, and nematode development was compromised in transgenic soybean hairy roots with a reduced expression of *GmCLV2, GmCRN*, or *GmCLV1*, indicating that soybean receptors GmCLV2/GmCRN and GmCLV1 play a critical important role in SCN parasitism.^11^ We previously characterized StCLV2 from potato, an Arabidopsis CLV2 orthologue. *StCLV2* was found to be highly upregulated at PCN infection sites and transgenic potato lines with reduced *StCLV2* expression showed increased resistance to PCN infection.^12^ StCLV2 was the only CLE receptor from potato that has been characterized and demonstrated to be directly involved in CLE signaling that is crucial for PCN to induce disease in potato.^12^ Parallel signaling pathways involving multiple CLE receptors are important for BCN and SCN to successfully infect their respective host plants.^20,21^ We thus hypothesized that additional CLE receptors from potato are likely involved in perceiving PCN CLE signals to promote successful nematode infection. In this study, we cloned and characterized *StRPK2* and *StCLV1* from potato, homologues of Arabidopsis *RPK2* and *CLV1* receptor genes, and investigated their role in PCN parasitism.

## Results and discussion

### Cloning and characterization of StRPK2 and StCLV1 genes from potato

**Figure 1.**
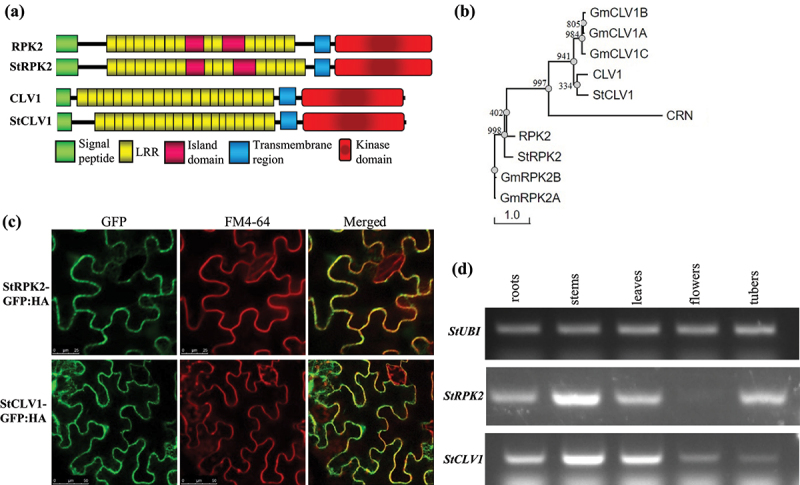
Characterization of StRPK2 and StCLV1. (a) Schematic representation of predicted domains in StRPK2 and StCLV1 in comparison with Arabidopsis RPK2 and CLV1, respectively. All these plant CLE receptors contain an N-terminal signal peptide, a LRR domain, a transmembrane domain, and a C-terminal kinase domain. (b) Phylogenetic relationship of StRPK2 and StCLV1 with their homologues from Arabidopsis and soybean (*Glycines max*). Multiple sequence alignment was obtained using the ClustalX program,^23^ and the unrooted consensus tree from 1,000 bootstrap replicates was generated using the PHYLIP 3.61 program.^24^ Sequences included are CLV1 (AT1G75820), RPK2 (AT3G02130), CRN (AT5G13290), *Glycine max* GmRPK2A, GmRPK2B, GmCLV1A, GmCLV1B, and GmCLV1C.^11^ (c) Subcellular localization of StRPK2 and StCLV1 in the plant cell. The *StRPK2-GFP:HA* or *StCLV1-GFP:HA* fusion construct was transiently expressed in *Nicotiana benthamiana* leaves, and GFP signals that colocalized with FM4-64 was observed in the plasma membrane. (d) RT-PCR analysis of *StRPK2* and *StCLV1* expression in various potato tissues. The potato *StUBI* gene (XM_006360024) was used as an internal control.

Searching the potato genome^22^ through BLAST analysis identified two potato genes Soltu.DM.03G007830.1 (named *StRPK2*; Genbank accession number OP171928) and Soltu.DM.04G036750.1 (named *StCLV1*; Genbank accession number OP171929), homologous to Arabidopsis *RPK2* and *CLV1*, respectively. *StRPK2* encodes an 1126-amino acid (aa) protein consisting of a predicted signal peptide, an extracellular domain of 16 LRRs, a transmembrane region, and a putative kinase domain located at the C-terminus (Figure 1a). StRPK2 is 76% similar and 64% identical to RPK2 from Arabidopsis. *StCLV1* encodes a 982-aa protein that contains a predicted signal peptide, an extracellular domain of 19 LRRs, a transmembrane region, and a C-terminal putative kinase domain (Figure 1a). The StCLV1 protein sequence is 76% similar and 63% identical to CLV1 from Arabidopsis. We further conducted phylogenetic analysis of these two receptors from Arabidopsis, potato, and soybean. The analysis revealed that StRPK2 and StCLV1 are more similar to their respective homologues from Arabidopsis than to those from soybean (Figure 1b). A recent study of LRR-RLK family proteins encoded by potato showed that StCLV1 we identified was grouped with CLV1 from Arabidopsis, rice, and tomato,^25^ further supporting our conclusion that StCLV1 is the orthologue of Arabidopsis CLV1.

To test the predicted membrane localization of StRPK2 and StCLV1, we transiently expressed an StRPK2-GFP:HA (hemagglutinin-tagged GFP) or StCLV1-GFP:HA fusion protein in *Nicotiana benthamiana* leaves. Both StRPK2-GFP:HA and StCLV1-GFP:HA were found to be accumulated predominantly at the cell periphery and colocalized with the styryl dye FM4-64 plasma membrane counterstain (Figure 1c).

The Arabidopsis *RPK2* and *CLV1* genes are expressed in multiple tissues including roots.^21,26,27^ We also examined the expression of *StRPK2* and *StCLV1* in various potato tissues using RT-PCR. Similar to *RPK2* and *CLV1, StRPK2* and *StCLV1* were found to be expressed in multiple potato tissues including roots although both genes showed the highest expression in the stem tissue that contained emerging branches in comparison with other tested tissues (Figures 1d and S1). Collectively, our data indicated that StCLV1 and StRPK2 may serve as functional receptors capable of perceiving PCN CLE peptides during nematode parasitism. Further studies, including confirming the binding of PCN CLE peptides to these receptors, are needed to verify this hypothesis.

### StRPK2 and StCLV1 have different spatial expression patterns in potato roots

**Figure 2.**
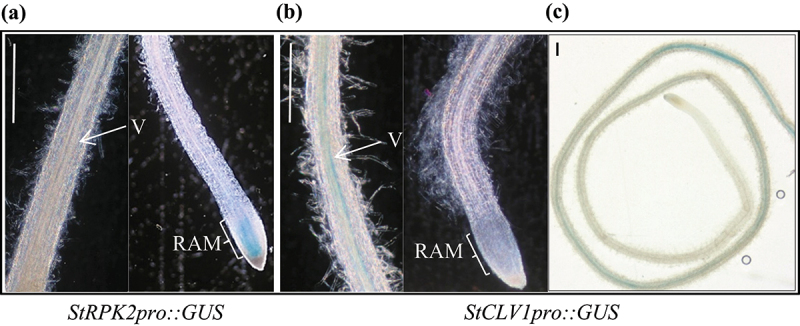
*StRPK2* and *StCLV1* show different spatial expression patterns in potato roots. Transgenic potato lines expressing *StRPK2pro::GUS* or *StCLV1pro::GUS* were generated and used to evaluate *StRPK2* and *StCLV1* expression in potato roots. *StRPK2pro::GUS* expression was observed primarily in the root apical meristem (a) whereas *StCLV1pro::GUS* expression was observed only in the root vascular tissue at the maturation region of potato roots under normal growth conditions (b,c). V, vasculature; RAM, root apical meristem. Bar = 1 mm.

Prior to this study, knowledge on the spatial expression of *StRPK2* and *StCLV1* in the potato root tissue was lacking. For StCLV1 and StRPK2 to function as receptors that perceive PCN-secreted CLE signals, they should be expressed in the appropriate root tissue during nematode infection. We utilized transgenic potato lines expressing a promoter::β-glucuronidase (GUS) construct, *StCLV1pro::GUS* or *StRPK2pro::GUS*, to investigate the expression of these two receptor genes in potato roots under normal growing conditions. In Arabidopsis roots, *CLV1* is expressed in the RAM and throughout the root vasculature starting at the maturation zone whereas *RPK2* is specifically expressed in the RAM.^21,26^ Through analyzing the promoter::GUS lines we found that *StCLV1* and *StRPK2* have different spatial expression patterns in potato roots. *StRPK2* appeared to be specifically expressed in the RAM whereas *StCLV1* only had expression in the vascular tissue at the maturation region of potato roots (Figure 2). The difference in root expression patterns between Arabidopsis *CLV1* and *StCLV1* might be due to the presence of different *cis*-acting elements identified in the promoter regions of *CLV1* and *StCLV1* (Table S1). *StRPK2* and *StCLV1* do not have overlapping expression regions. Thus, it would be interesting to determine whether these two receptors have similar or different functions in potato roots.

### StRPK2 but not StCLV1 is responsive to PCN infection

**Figure 3.**
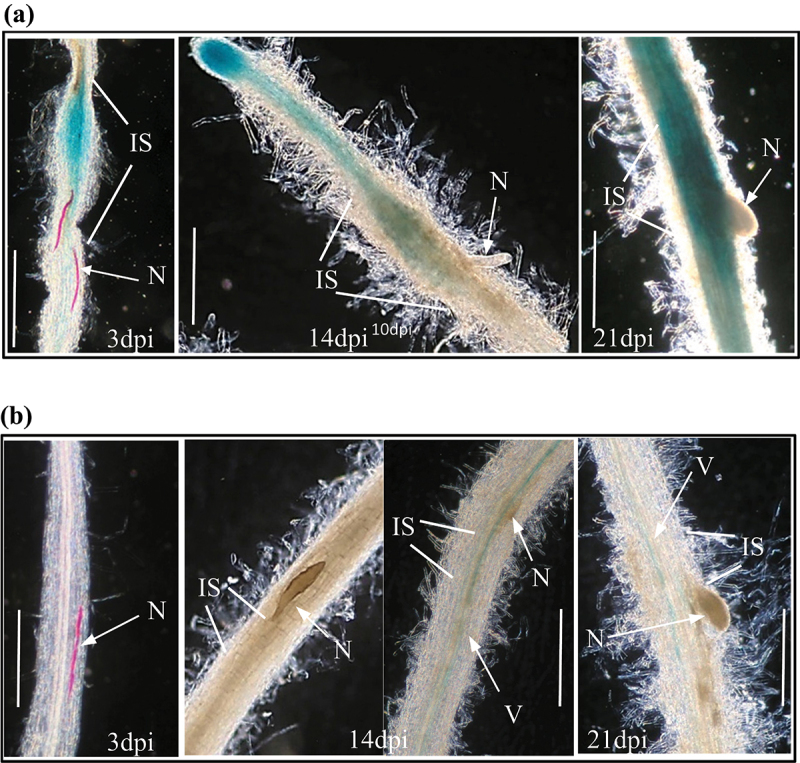
*StRPK2* but not *StCLV1* is responsive to nematode infection. Transgenic potato lines expressing *StRPK2pro::GUS* or *StCLV1pro::GUS* were generated and used to determine *StRPK2* or *StCLV1* expression during nematode infection. (a) Roots of transgenic potato lines expressing *StRPK2pro::GUS* were infected with the potato cyst nematode (*Globodera rostochiensis*), and GUS activity was observed at nematode infection sites associated with parasitic second-stage juveniles (J2) (at 3 days post-inoculation, dpi) (left), third-stage juveniles (at 14 dpi) (middle), and fourth-stage juveniles (at 21 dpi) (right). (b) Roots of transgenic potato lines expressing *StCLV1pro::GUS* were infected with the potato cyst nematode (*Globodera rostochiensis*), but no obvious GUS activity was detected at nematode infection sites associated with parasitic J2 (at 3 dpi) (left), parasitic J3 (at 14 dpi) (middle), and parasitic J4 (at 21 dpi) (right). N, nematode; IS, infection site; V, vasculature. Bar = 1 mm.

To determine whether *StRPK2* and *StCLV1* are responsive to nematode infection, transgenic *StRPK2pro::GUS* and *StCLV1pro::GUS* lines were infected with PCN and monitored during nematode development. For the *StRPK2pro::GUS* lines, strong GUS expression was detected at and around nematode infection sites as soon as the second-stage juveniles began to feed, and the expression level remained high at the infection sites associated with parasitic nematodes at later developmental stages (Figure 3a). GUS expression was also observed in the vasculature adjacent to nematode infection sites (Figure 3a). The induced expression of *StRPK2* at nematode infection sites indicated that StRPK2 may be involved in perceiving PCN CLE peptides during nematode infection. We previously showed that *StCLV2* is also upregulated at nematode infection sites.^12^ PCN *CLE* genes are expressed throughout nematode parasitic stages.^8^ The continuous secretion of CLE effectors might allow the nematode to activate and interact with host endogenous CLE receptors including StCLV2 and StRPK2 to promote syncytium formation and successful nematode parasitism. For the *StCLV1pro::GUS* lines, however, almost no GUS expression was observed at nematode infection sites over the course of nematode development (Figure 3b), indicating that StCLV1 may not have role in PCN parasitism.

### Silencing StRPK2 reduces PCN infection

**Figure 4.**
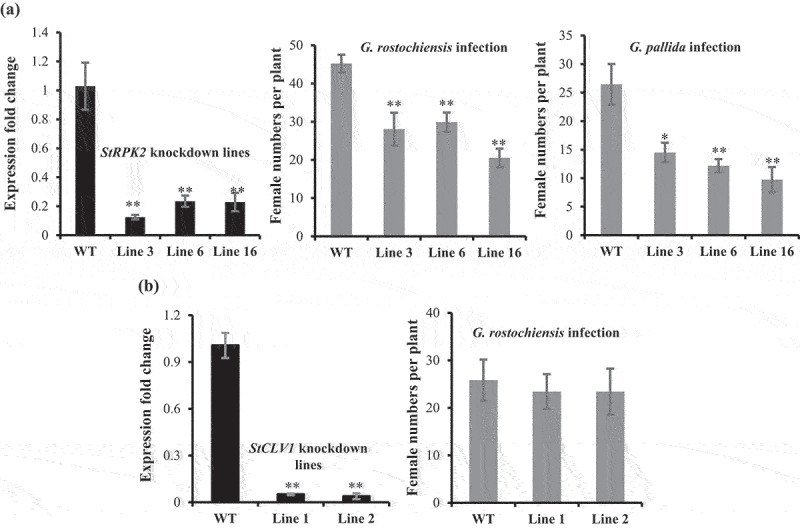
*StRPK2* but not *StCLV1* plays an important role in nematode parasitism. Transgenic potato lines expressing an artificial microRNA construct targeting *StRPK2* or *StCLV1* were generated. Quantitative RT-PCR was used to evaluate *StRPK2* or *StCLV1* expression in the obtained transgenic lines. Three independent *StRPK2* knockdown lines (lines 3, 6, and 16) and two independent *StCLV1* knockdown lines (lines 1 and 2) were found to have dramatically reduced expression of the target gene compared with the wild-type (WT) plant. Data represents means ± SD of three biological replicates, normalized to the potato *StUBI* gene (XM_006360024) and relative to expression in the WT plant (**P < .001, Student’s *t* test) (a: left; b: left). (a) *StRPK2* knockdown lines showed reduced susceptibility to nematode infection. Wild-type and transgenic lines (20 vegetatively propagated plantlets for each line) were inoculated with *Globodera rostochiensis* or *G. pallida* juveniles, and nematode females were counted at 5 weeks after nematode inoculation. Values are means ± SD of two independent experiments (*0.01 < *P* < .05, **P < .01, Student’s *t* test). (b) *StCLV1* knockdown lines showed a nematode susceptibility level similar to that of the WT plants. WT and *StCLV1* knockdown lines (7-9 vegetatively propagated plantlets for each line) were inoculated with *G. rostochiensis* juveniles, and nematode females were counted at 5 weeks after nematode inoculation. Values are means ± SD (*n* = 7–9). Similar results were obtained from two independent experiments and one representative experiment is shown. No statistical difference was observed between the *StCLV1* knockdown lines and the WT plants.

To further investigate a role of *StRPK2* and *StCLV1* in nematode parasitism, we made artificial microRNA constructs specifically targeting *StRPK2* and *StCLV1* and transformed them individually into potato. Screening the obtained independent transgenic lines identified two to three lines where the expression of the targeted gene was dramatically reduced in roots compared with wild-type potato lines (Figure 4, a and b). *StRPK2* and *StCLV1* knockdown lines did not show obvious phenotypic difference in roots and aboveground tissues in comparison with the control plants. We then tested nematode infection on *StRPK2* and *StCLV1* knockdown lines along with wild-type plants. At 35 days after nematode infection, nematode females formed on the roots were counted. Our results showed that the three *StRPK2* knockdown lines were more resistant to infection by both PCN species as the number of nematode females recovered from the three lines was approximately 36% to 62% less than those from the control lines (Figure 4a). The decreased susceptibility to PCN supports a role of *StRPK2* in nematode parasitism. As for the *StCLV1* knockdown lines, although a reduced number of nematode females was observed, our statistical analysis revealed that the female number observed on the *StCLV1* knockdown lines was not significantly different from that observed on the control lines (Figure 4b). Meanwhile, *StCLV1* was found not to be responsive to nematode infection. Collectively, our results indicated that *StCLV1* may not have a role in PCN parasitism.

## Conclusion

Previously, we reported that in the potato-PCN pathosystem, nematode CLE signaling through StCLV2 is required for successful nematode parasitism.^12^ In this study, we characterized two candidate CLE receptors from potato, StCLV1 and StRPK2, and determined that StRPK2 but not StCLV1 is important for PCN parasitism. *StRPK2* knockdown lines showed enhanced resistance to both PCN species, thus targeting *StRPK2* may represent a viable means for generating potatoes with broad-spectrum nematode resistance. PCN encodes multidomain CLE effectors, which likely give rise to multiple different bioactive CLE peptides when delivered into potato roots during nematode infection.^12^ Therefore, it is highly likely that more members of the LRR-RLK family in potato are involved in perceiving and relaying PCN-secreted CLE signals. The GrCLE1 peptide from PCN was previously shown to be able to bind Arabidopsis BAM1 and BAM2 receptors.^28^ Guo et al. (2017) recently identified the expression of B-type *CLE* genes from SCN and BCN.^29^ The study further revealed that the TDIF (tracheary element differentiation inhibitory factor)-TDR (TDIF receptor)-WOX4 pathway, which promotes procambial meristem cell proliferation, is involved in BCN parasitism on Arabidopsis.^29^ B-type CLEs have not been identified in PCN. Further studies are needed to determine if any of the BAM and TDR homologues from potato and soybean have a role in PCN and SCN parasitism, respectively. Meanwhile, it would be interesting to investigate if there is any connection among CLE signaling pathways mediated by different CLE receptors during nematode infection. Cyst nematode secreted-CLE effectors are the key signaling molecules crucial to nematode parasitism. Identification of host receptors targeted by nematode CLE peptides as well as the associated downstream signaling components regulated by host receptors may provide novel means to engineer nematode resistance in economically important crops including potato.

## Materials and methods

### Nematode culture and plant materials

Maintenance of the potato cyst nematode (*Globodera rostochiensis* and *G. pallida*) culture and nematode inoculation on cultured potato plantlets were conducted as described previously.^30,31^ Potato (*Solanum tuberosum*) cv. Désirée was used for generating transgenic plants and *Nicotiana benthamiana* was used for transient expression assays.

### Sequence and phylogenetic analysis of StRPK2 and StCLV1

Arabidopsis *RPK2* and *CLV1* gene sequences were used to search the potato genome via BLAST analysis to identify the respective homologous sequences from potato and then named them as *StRPK2* and *StCLV1*, respectively. The SMART (simple modular architecture research tool) database (http://smart.embl-heidelberg.de/) and NCBI-CDD (http://www.ncbi.nlm.nih.gov/Structure/cdd/wrpsb.cgi) were used to identify the signal peptide, the transmembrane region, and LRR and kinase domains in *Soltu.DM.03G007830.1* (named *StRPK2*) and *Soltu.DM.04G036750.1* (named *StCLV1*) encoded protein sequences. Phylogenetic analysis of Arabidopsis RPK2 and CLV1 and their homologues from potato and soybean was conducted as described previously.^30^ Full-length protein sequences were used for the analysis.

### Characterization of the CLV1 promoter region

The PlantCARE database (http://bioinformatics.psb.ugent.be/webtools/plantcare/html/)^32^ was used to identify *cis*-acting elements in the Arabidopsis *CLV1* (−3450 bp to −1 bp) and *StCLV1* (−3465 bp to −1 bp) promoter regions (Table S1).

### Subcellular localization

The *StRPK2* (OP171928) and *StCLV1* (OP171929) genes were identified in the annotated potato genome sequence and amplified from complementary DNA derived from potato roots using primer pairs StRPK2_ATGF and StRPK2_TGAR, and StCLV1_ATGF and StCLV1_TGAR (Table S2). The amplified PCR products were cloned into the pBIN61-GFP:HA binary vector^33^ at the X*ba*I and B*am*HI sites to generate *StRPK2-GFP:HA* and *StCLV1-GFP:HA* fusion protein constructs. *Agrobacterium tumefaciens* strain C58C1 transformed with *StRPK2-GFP:HA* or *StCLV1-GFP:HA* was infiltrated into *N. benthamiana* leaves.^31^ Two days after infiltration, leaves were collected and stained in FM4-64 solution (Invitrogen). Leaf sections were then visualized with an SP5 Leica confocal microscope.

### Histochemical GUS assay and nematode staining

A 2484-bp DNA sequence upstream of the start codon of *StRPK2* that likely covers all the *cis*-regulatory elements of the gene and a 3465-bp DNA sequence upstream of the start codon of *StCLV1* containing all the possible *cis*-regulatory elements were amplified from potato genomic DNA by PCR using primer pairs StRPK2pr_SalI-2484 F and StRPK2pr_BamHI-2 R for *StRPK2* and StCLV1_SalI_-f3465F and StCLV1_BamHI_R for *StCLV1* (Table S2). The amplified DNA fragments were cloned into the binary vector pBI101.2^34^ at the *Sal*I and *Bam*HI sites to generate the *StRPK2pro::GUS* and *StCLV1pro::GUS* constructs. Transgenic potato lines expressing the individual promoter-GUS construct were generated as described.^31^ Nematode infection on transgenic potato plantlets as well as nematode staining and histochemical GUS assays were performed as described previously.^12^

### Generation of gene knockdown lines and nematode infection assays

To make the artificial microRNA (amiRNA) construct, a 21-mer sequence (5’-TAATACATGGCACGATTGCCG-3’) targeting *StRPK2* and a 21-mer sequence (5’- TATCTGACTATATCTGCGCCG-3’) targeting *StCLV1*, designed according to the Web artificial microRNA designer interface WMD3 (wmd3.weigelworld.org), was engineered into the miR319a precursor by replacing the endogenous MIR319a sequence in pRS300^35^ using overlapping PCR^36^ with primers as listed in Table S2. The new amiR319a precursor was then cloned into the modified binary vector pSMD1 at the *Xho*I and *Sac*I sites to make the *pSMD1-StRPK2ami* and the *pSMD1-StCLV1ami* artificial microRNA constructs.^12^ These constructs were transformed individually into *A. tumefaciens* strain LBA4404 and used for potato transformation. Transgenic potato lines expressing the amiRNA constructs were generated as described.^31^ Nematode infection assays on transgenic potato plantlets were conducted and the number of nematode females was counted on each plantlet at 5 weeks after nematode inoculation.^30,31^ The experiments were conducted at least two times with a minimum of 15 replicates for each line, and similar results were obtained. The results were analyzed by the student *t*-test.

### Reverse transcription-PCR and quantitative reverse transcription-PCR

mRNA from roots, leaves, and stems of cultured potato plantlets and flowers and tubers from a potted potato plant was extracted and used to determine *StRPK2* and *StCLV1* expression in various potato tissues by RT-PCR. mRNA from roots of cultured plantlets of transgenic potato lines expressing *pSMD1-StRPK2ami* or *pSMD1-StCLV1ami* as well as a wild-type control plant was extracted and used to quantify *StRPK2* and *StCLV1* expression by quantitative RT-PCR as described previously.^30^ mRNA was treated with DNase I and confirmed with no genomic DNA contamination by PCR before cDNA synthesis. The potato *StUBI* gene (XM_006360024) was used as an endogenous reference. Primers used are listed in Table S2. The RT-qPCR data were obtained from three independent experiments with three technical replicates for each cDNA sample.
